# Post-operative atrial fibrillation, intraoperative autologous blood salvage and transfusion in cardiac surgery

**DOI:** 10.1016/j.htct.2026.106467

**Published:** 2026-04-26

**Authors:** Sergio Domingos Vieira, Fernanda da Cunha Vieira, Leandro Felipe Figueiredo Dalmazzo, Fabiana Akil, Leila Patrícia de Sousa Fontenele, Aline Miranda de Souza, Ana Paula Carrijo Rodrigues, Sanny Marcele da Costa Lira, Viviani de Lourdes Rosa Pessoa, Shirley Lopes de Castilho

**Affiliations:** Grupo Gestor de Serviços de Hemoterapia (Grupo GSH), São Paulo, SP, Brazil

**Keywords:** Atrial fibrillation, Operative blood salvage, Cardiac surgical procedures, Blood transfusion

## Abstract

**Background and objectives:**

Postoperative atrial fibrillation is the most common complication following cardiac surgical procedures. The objective of this study was to assess a possible association of postoperative atrial fibrillation, intraoperative autologous blood salvage, and blood transfusion.

**Methods:**

A total of 357 adult patients undergoing cardiac surgery with the use of intraoperative autologous blood salvage were included. They were divided into two groups: one with 161 patients (45%) who only received their recovered autologous blood, and another with 196 patients (55%) who additionally required the transfusion of homologous blood components. Demographic data, pre- and post-operative laboratory parameters, transfusion profiles, and clinical outcomes (mortality) were compared.

**Results:**

The number of recovered autologous blood units was not associated with an increased risk of postoperative arrhythmia (Odds ratio: 0.75; 95% CI: 0.45–1.11). Intraoperative homologous blood transfusion was associated with a significantly increased risk of death (Hazard ratio: 5.17; 95% CI 1.44–18.56). When autologous salvaged blood volume and homologous components were evaluated together, homologous transfusion was associated with higher risk of postoperative atrial fibrillation (Odds ratio: 2.51, 95% CI 1.18–5.65), whereas number of autologous units was not (OR 0.66, 95% CI 0.39–1.01). Transfusion at any time point (intraoperative, intensive care unit [ICU], or ward) was also associated with an increased risk of death (Hazard ratio: 5.15, 95% CI 1.16–22.94).

**Conclusion:**

While advanced age was significantly associated with postoperative atrial fibrillation, no association was found with intraoperative autologous blood salvage and blood transfusion.

## Introduction

Postoperative atrial fibrillation (POAF) is the most common complication following cardiac surgical procedures [1]. Its incidence ranges from 20% to 50%, depending on factors such as the type of surgery, patient population, and diagnostic criteria used [2,3]. According to data from the Society of Thoracic Surgeons (STS) Adult Cardiac Surgery Database, POAF occurs in 25% of patients undergoing isolated coronary artery bypass grafting (CABG), 30% of those undergoing isolated valve procedures, and 40% to 50% of those undergoing combined CABG/valve surgeries [4].

In the United Kingdom, approximately 32,000 adult patients across 35 centers undergo cardiac surgery annually, and 30% develop POAF. Even a single episode of POAF extends ICU stay by 12–24 h and hospital stay by 2–5 days [5,6], and nearly triples in-hospital mortality (from 0.5% to 3.3%) and one-year mortality (from 3.7% to 9.9%) [7,8]. POAF increases healthcare costs by approximately £10,000 ($12,500) per patient in the first postoperative year [9]. For these reasons, the prevention of POAF was identified as one of the top ten research priorities by the James Lind Alliance Cardiac Surgery Priority Setting Partnership in 2019, involving patients, caregivers, and healthcare professionals [10].

Multiple studies have confirmed that patients who develop POAF face increased risks of morbidity, mortality [11,12], and complications such as stroke, heart failure, renal dysfunction, and prolonged hospitalization [12,13]. Reducing its incidence may improve clinical outcomes, increasing interest in identifying risk factors that could enable the implementation of preventive strategies.

The pathophysiology of new-onset POAF remains poorly understood. Recent evidence supports an inflammatory mechanism in its development [14,15]. Transfusions of red blood cells (RBCs) or whole blood (WB) modulate the inflammatory response to cardiac surgery by altering various inflammatory mediators and enhancing systemic inflammation [16,17]. Currently, the most effective and safest method to reduce the need for transfusion and its potential complications is intraoperative autologous blood salvage, especially in cardiac surgeries (Class I; Level A evidence) [18]. This technique is a cornerstone of patient blood management [19]. Autologous cell salvage may improve red blood cell viability, preserve their biconcave shape, enhance oxygen delivery, and attenuate the inflammatory response to surgery [20,21].

This retrospective cohort study was designed to assess a potential association between POAF, autologous blood salvage, and transfusion as the primary outcome, and mortality risk as a secondary outcome.

## Material and methods

This retrospective observational cohort study collected data from 26 hospitals in four Brazilian states, evaluating 357 adult patients who underwent cardiac surgery with intraoperative autologous blood salvage between January 2021 and December 2023.

The study was conducted in accordance with the Declaration of Helsinki (2008 revision). Due to the retrospective nature of the study and the use of anonymized data, consent was considered unnecessary.

Nearly all types of cardiac surgeries were represented: CABG, valve replacement or repair (VR), aortic aneurysm repair (AAR), the Bentall-de Bono procedure (BB), redirection of blood flow (RBF), myectomy, cardiac tumor resection, and combined procedures (e.g., CABG + VR, CABG + AAR, or VR + AAR). Preoperative and intraoperative variables (volume and number of autologous units recovered) were assessed for potential associations with an increased risk of transfusion.

### Cell saver protocol

All cell salvage machines were calibrated and programmed according to a specific protocol based on patient blood volume, with bowl size and flow rates adjusted accordingly for each cycle phase. Blood was collected from skin incision through wound closure using a dual-lumen suction catheter designed for blood collection. Suction pressure was maintained below 100 mmHg to minimize hemolysis. Anticoagulation was achieved using 25,000 IU of heparin diluted in 1000 mL of 0.9% saline solution. Anticoagulant flow was adjusted based on surgical field bleeding rates, and the blood was filtered and stored in a cardiotomy reservoir. Whenever possible, residual blood from the cardiopulmonary bypass circuit was also redirected to the reservoir to maximize autologous blood salvage.

The cell saver system operated in either manual or automatic mode, with automatic mode prioritized to ensure standardized, pre-defined blood processing with minimal operator intervention. Red blood cells were washed and resuspended in 0.9% saline, resulting in a hematocrit of approximately 60%. These washed autologous red blood cells were transferred to a sterile reinfusion bag and transfused intraoperatively.

In the perioperative period, patients requiring additional blood volume beyond their own autologous blood received leukoreduced homologous red blood cell transfusions. In cases of excessive blood loss and cardiovascular instability, transfusions were administered at the discretion of the anesthesiology or intensive care team.

### Statistical analysis

Data are presented as mean ((standard deviation), median (interquartile range [IQR]), or frequency (valid %) as appropriate. Comparisons between groups were performed using Student’s *t*-test, Mann-Whitney U test, or chi-square test, depending on data distribution and normality assessed via the Shapiro-Wilk test. Logistic regression models and Cox proportional hazards models were used to evaluate associations between variables of interest and postoperative arrhythmia and mortality, respectively. All analyses were conducted using R statistical software (R Foundation for Statistical Computing, 2024).

## Results

Patients were divided into two groups: one with 161 patients (45%) who only received their recovered autologous blood, and the other with 196 patients (55%) who also required transfusion of homologous blood components (Table 1). Patients who received homologous blood were more frequently female (p-value <0.001), had lower body weight (p-value = 0.002), and exhibited lower preoperative levels of hemoglobin, hematocrit, and platelets (p-value <0.001).

**Table 1:** Patient characteristics (n = 357).

Variable	
Age (years) – mean (SD	58.7 (13.4
Male - n (%)	263 (73.7)
Weight (kg) – mean (SD	78.9 (15.3
State of origin - n (%)	
Bahia	12 (3.4)
Distrito Federal	13 (3.6)
Rio de Janeiro	28 (7.8)
São Paulo	304 (85.2)
Platelets (1000/mm^3^) – mean (SD	210.3 (68.4)
Hematocrit (%) – mean (SD	38 (6.6)
Hemoglobin (g/dL) – mean (SD	12.6 (2.3)
Kit (bowl) - n (%)	
55 mL	1 (0.3)
125 mL	35 (9.8)
225 mL	321 (89.9)
pRBC (mL) – mean (SD	556.7 (390.8)
Units salvaged/patient – mean (SD	1.43 (1.0)
Atrial Fibrillation/Arrythmia - n (%)	37 (10.4)
Transfusion - n (%)	196 (54,9)

SD: Standard deviation; pRBC: packed red blood cells.

Regarding autologous blood recovery, the median volume was 505 mL (1.3 units) in the group that required homologous transfusion, compared to 462 mL (1.2 units) in the group that did not require homologous components (p-value <0.001) (Table 2).

**Table 2:** Patient characteristics per group.

	Homologous blood		P-value
	No (n = 161)	Yes (n = 196)	
Age (years) – median (IQR)	59 (15)	62 (20)	0.243
Female – n (%)	26 (16.1)	68 (34.7)	**<0.001**
Weight (kg) – median (IQR)	80 (18)	77 (22)	**0.005**
Hemoglobin (g/dL) – median (IQR)	13.4 (2.15)	12 (3.2)	**<0.001**
Hematocrit (%) – median (IQR)	40 (6.7)	37 (8.75)	**<0.001**
Platelets (x10^9^/L) – median (IQR)	215 (68)	200 (72)	**0.047**
Kit (bowl) – n (%)			0.63
55 mL	0	1 (0.5)	
125 mL	15 (9.3)	20 (10.2)	
225 mL	146 (90.7)	175 (89.3)	
pRBC (mL) – median (IQR)	462 (248)	505 (365.75)	**<0.001**
Autologous units recovered – median (IQR)	1.2 (0.6)	1.3 (1.0)	**<0.001**

IQR: interquartile range; pRBC: packed red blood cells.

The most commonly used blood component in the operating room was packed red blood cells (PRBCs; n = 107), with a median of 2 (IQR = 1.5) units. PRBCs were also the most frequently used component in the ICU.

With respect to surgical type, 163 patients (45.7%) underwent CABG, 129 (36.1%) underwent valve surgery, and 34 (9.5%) underwent aortic aneurysm repair. Other surgical procedures were less frequent.

Autologous blood transfusion was not associated with POAF (Odds ratio [OR] = 0.75; 95% CI: 0.45–1.11). The only factor independently associated with POAF in the multivariate analysis was older age (OR 1.04; 95% CI: 1.01–1.08) (Table 3).

**Table 3:** Blood transfusion and postoperative atrial fibrillation.

	OR	95% CI	P-value
***Univariable***			
Homologous transfusion	1.13	0.49–2.62	0.77
Autologous units recovered	0.75	0.45–1.11	0.22
***Multivariable***			
Homologous transfusion	1.02	0.40–2.61	0.96
Age	1.04	1.01–1.08	**0.02**
Male	0.52	0.16–1.73	0.29
Weight	1.01	0.98–1.03	0.63
Preoperative hemoglobin	1.00	0.83–1.20	0.99

Logistic regression analyses were conducted. Covariates included in the multivariable analyses were selected based on biological plausibility (age) and baseline group imbalances (others).

OR: odds ratio; CI: confidence interval.

When autologous salvaged blood volume and homologous components were evaluated together, homologous transfusion was associated with higher risk of POAF (OR 2.51; 95% CI 1.18–5.65), whereas number of autologous units was not (OR 0.66; 95%CI 0.39–1.01). This analysis was adjusted for pre-operative hemoglobin (Table 4).

**Table 4:** Simultaneous evaluation of homologous transfusion and autologous transfusion regarding the odds of post-operative atrial fibrillation.

	OR	95% CI	P-value
Homologous transfusion	2.51	1.18–5.65	**0.02**
Autologous units salvaged	0.66	0.39–1.01	0.09
Preoperative hemoglobin	0.99	0.85–1.16	0.92

A multivariable logistic regression analysis was conducted to evaluate the mutual influence of homologous and autologous blood transfusion, adjusted for baseline preoperative hemoglobin.

OR: odds ratio; CI: confidence interval.

Regarding mortality, intraoperative homologous blood transfusion was associated with a significantly increased risk of death (HR: 5.67; 95% CI: 1.64–19.62). This association remained consistent in the multivariate analysis (HR: 5.17; 95% CI: 1.44–18.56), adjusted for age, sex, preoperative hemoglobin, and body weight (Table 5). Transfusion at any time point (intraoperative, ICU, or ward) was also associated with an increased risk of death (HR: 5.15; 95% CI: 1.16–22.94).

**Table 5:** Homologous blood transfusion and mortality.

	HR	95% CI	P-value
**Univariable**			
Intraoperative homologous transfusion	5.67	1.64 – 19.62	**<0.01**
Any homologous transfusion	5.89	1.35–25.65	**0.02**
**Multivariable**			
Intraoperative homologous transfusion	5.17	1.44–18.56	**0.01**
Age	1.03	0.99–1.07	0.11
Male	1.29	0.44–3.74	0.64
Weight	0.99	0.96–1.02	0.71
Baseline hemoglobin	0.90	0.76–1.07	0.25
Any homologous transfusion	5.15	1.16–22.94	**0.03**
Age	1.03	0.99–1.07	0.11
Male	1.14	0.39–3.29	0.81
Weight	0.99	0.96–1.03	0.74
Baseline hemoglobin	0.88	0.74–1.05	0.16

Cox proportional odds regression analyses were conducted. Covariates included in the multivariable analyses were selected based on biological plausibility (age) and baseline group imbalances (others).

Analyses were conducted for patients who received homologous blood products in the operating room (intraoperative) and at any time during hospital stay (any), including operating room, intensive care. and ward.

HR: hazard ratio; CI: confidence interval.

**Fig. 1:**
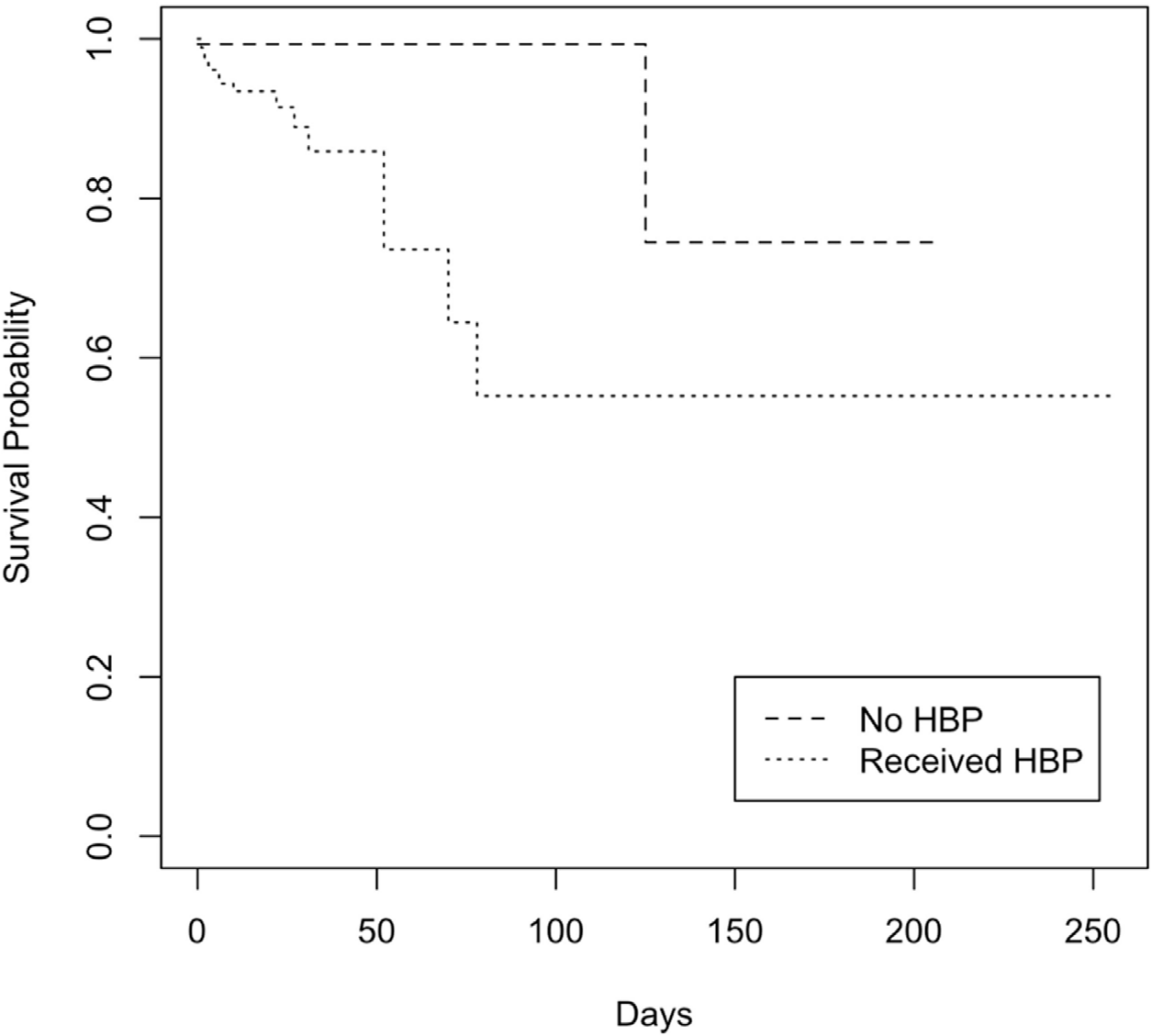
Survival analysis stratified according to use of homologous blood products Patients who received homologous blood products (HBP) had lower survival (log-rank - p-value <0.01).

Both a higher volume of autologous blood recovered and the use of homologous blood transfusion were associated with an increased risk of death. The number of autologous units recovered was associated with a higher risk of mortality (HR: 1.83; 95% CI: 1.39–2.41), as was older age (HR: 1.04; 95% CI: 1.01–1.08). Conversely, higher preoperative hemoglobin levels were associated with a reduced risk of death (HR: 0.80; 95% CI: 0.67–0.96) (Table 6). Patients who needed homologous blood transfusion had lower survival (log-rank - p-value <0.01, Figure 1).

**Table 6:** Autologous units salvaged and mortality.

	HR	95% CI	P-value
**Univariable**			
Salvaged units	1.46	1.19–1.80	**<0.01**
**Multivariable**			
Salvaged units	1.83	1.39–2.41	**<0.01**
Age	1.03	1.00–1.07	**0.04**
Male	1.07	0.36–3.13	0.90
Weight	0.98	0.96–1.02	0.34
Baseline hemoglobin	0.80	0.67–0.96	**0.01**

Cox proportional odds regression analyses were conducted. Covariates included in the multivariable analyses were selected based on biological plausibility (age) and baseline group imbalances (others).

HR: hazard ratio; 95% CI: confidence interval.

## Discussion

The transfusion-related findings in this study underscore the urgent need to implement Patient Blood Management, as emphasized by the World Health Organization (WHO) in 2021. The WHO highlighted that anemia has a global prevalence, affecting billions of individuals worldwide. Preoperative anemia in surgical patients is more prevalent than in the general population and is associated with significantly increased morbidity, mortality, and hospital length of stay across most surgical procedures [22].

When comparing baseline characteristics between groups, preoperative hematocrit, hemoglobin, and platelet levels indicated that the group requiring homologous blood transfusions already presented with anemia at admission (hematocrit median = 37%; IQR: 8.755 and hemoglobin median = 12 g/dL; IQR: 3.2 g/dL; p-value <0.001). In this study, the risk-adjusted impact of preoperative hematocrit on morbidity and mortality is consistent with prior publications in cardiac surgery patients. Multiple studies have demonstrated a significant correlation between preoperative or intraoperative anemia and adverse cardiac surgical outcomes [23, 24, 25].

LaPar et al. investigated the relative contribution of preoperative hematocrit and packed red blood cell (PRBC) transfusion to postoperative outcomes following CABG. In this multi-institutional analysis of over 33,000 patient records, the key findings showed that PRBC transfusions had a greater impact than preoperative hematocrit on postoperative mortality, stroke, and renal failure [26]. Therefore, transfusion may be more of a marker of a critically ill patient than the direct cause of death.

In the present cohort, the anemic group also had significantly lower platelet counts (p-value = 0.047), which may explain the higher incidence of reported ‘increased intraoperative bleeding’ (20 patients – 10.2%), the need for homologous blood components in the operating room (158 patients – 80.6%), and the higher volume of recovered autologous red blood cells (median: 505 mL; IQR: 365.75 mL - p-value <0.001). In contrast, this complication was reported in only two patients (1.2%) in the non-transfused group.

Some authors have suggested that perioperative blood transfusions may induce systemic inflammatory changes, potentially increasing the likelihood of developing POAF in transfused patients during cardiac surgery [14,27,28]. However, the findings of this study are supported by a previous study that showed that intraoperative autologous blood transfusion was not associated with POAF (OR: 1.13; 95% CI: 0.49–2.62), and the number of autologous blood units recovered was also not associated with increased risk of POAF (OR: 0.75; 95% CI: 0.45–1.11) [29]. These results remained consistent after adjustment for age, weight, sex, and preoperative hemoglobin level.

### Limitations

The main limitations of this study are its observational, non-randomized design and the relatively small patient cohort. As a multi-institutional study involving diverse surgical, anesthetic, perfusion, and intensive care teams, the results should be interpreted with caution due to the high degree of inter-team heterogeneity. Furthermore, the clinical significance and long-term implications of POAF in cardiac surgery were not evaluated.

## Conclusion

The findings of this multi-institutional retrospective cohort study of cardiac surgical patients indicate no association between POAF, intraoperative autologous blood recovery, and blood transfusion. The only factor associated with POAF was advanced age. Preoperative anemia and blood transfusion independently increased the risk of postoperative morbidity and mortality. Urgent implementation of patient blood management strategies is warranted to improve surgical outcomes.

## Ethical considerations

All procedures performed in studies involving human participants were in accordance with the ethical standards of the institutional research committee and with the 1964 Helsinki Declaration and its later amendments.

## Funding

This research did not receive any specific grant from funding agencies in the public, commercial, or not-for-profit sectors.

## Data availability

The data that support the findings of this study are available from the corresponding author upon reasonable request.

## Conflicts of interest

The author declares no conflicts of interest.
